# Entozoa in Veal and Beef

**Published:** 1865-05

**Authors:** T. Spencer Cobbold

**Affiliations:** Middlesex Hospital


### ENTOZOA IN VEAL AND BEEF.

To the Editor of The Lancet.---*Sir:*---At the risk of riding the "hobby-horse" a little to fast, I would call the attention of your readers to an interesting entozoological fact.

Professor Simonds and myself have just succeeded in rearing the larvæ of *tœnia mediocanellata* in a calf, but at present the cysticerci are not perfectly developed. We know, however, that the larvæ are there, not only by the symptoms produced, but also from ocular demonstration; for having removed a very small portion of the right sterno-cleido-mastoid muscle (weighing 22 grains), three minute cysticercus-vesicles were discovered in the portion detached. Even if the other muscles (to say nothing of the heart, lungs, and liver) are affected to the same extent only, we shall eventually find not less than 30,000 larval entozoa in this one animal---enough to give 30,000 persons the *tœnia mediocanellata*, should they be severally disposed to swallow a cysticercus.

By a *post hoc propter hoc* kind of reasoning, we might say that the little operation has done good, for the calf in certainly much better since the removal of three of its "guests." Each larva is about the size of a pin's head.

Now that I am on the subject of entozoa, perhaps you will allow me to correct an erroneous and widely-spread impression which seems to have taken firm hold on the public mind. It is supposed that I am averse to "sewage-distribution" on entozoological grounds. This is an error. The object of my "pamphlet" is to show that, *without due precautions* (based upon an intelligent appreciation of the facts which recent helminthological discoveries have unfolded), very serious evils may result.

The majority close their senses against all evidence, and vote science a nuisance, whilst the more enlightened minority demand proof from the past, and point to Edinburgh experiences. To the latter class I would say that the case of the Craigentinny-meadows affords negative evidence, simply because the cows fed upon their grass-produce are employed to give milk, and are not commonly eaten as food. There are other reasons, upon which I cannot now enlarge; and as to the *Bilharzia*, that must also, for the present, stand apart.

I am not aware that I have advanced any statements that are inconsistent either with truth or honesty of purpose, and it has yet to be shown that the conclusions which I have drawn are at variance with the principles of sound deductive reasoning. It may serve an agriculturist's turn to characterize my views as those of an "alarmist," but after the able article which has appeared in your pages, I can well afford to "rest and be thankful." I am Sir, your obedient servant,

T. SPENCER COBBOLD, M.D., F.R.S.

Middlesex Hospital, Feb. 14th, 1865.

